# Diabetic Neuropathy and Minimum Effective Anesthetic Concentration of Mepivacaine for Axillary Brachial Plexus Block: A Prospective Observational Study

**DOI:** 10.3390/jpm14040353

**Published:** 2024-03-27

**Authors:** Na-Eun Kim, Woo-Joo Lee, Jong-Kwon Jung, Jang-Ho Song, Kyung-Lim Joa, Chun-Woo Yang, Eui-Chan Jung, Soo-Man Jo, Yeong-Seung Ko

**Affiliations:** 1 The Department of Anesthesiology and Pain Medicine, School of Medicine, Inha University, 100 Inha-ro, Michuhol-gu, Incheon 22212, Republic of Korea; 220241@inha.ac.kr (N.-E.K.); ckchung@inha.ac.kr (J.-K.J.); jhs@inha.ac.kr (J.-H.S.); ecjung0506@inhauh.com (E.-C.J.); ssoosoo1@inhauh.com (S.-M.J.); ysko@inhauh.com (Y.-S.K.); 2 The Department of Public Health Science, Seoul National University, 1 Gwanak-ro, Gwanak-gu, Seoul 08826, Republic of Korea; lwj221@snu.ac.kr; 3 The Department of Physical and Rehabilitation Medicine, School of Medicine, Inha University, 100 Inha-ro, Michuhol-gu, Incheon 22212, Republic of Korea; drjoakl@inha.ac.kr

**Keywords:** brachial plexus, concentration, diabetics, local anesthetic, nerve block, neuropathy

## Abstract

Nerves in patients with diabetic neuropathy (DN) show increased susceptibility to local anesthetics, potentially requiring a decreased dose. We investigated whether the minimum effective anesthetic concentration (MEAC) of mepivacaine for successful axillary block is lower in patients with DN than in those without. This prospective observational study included patients with DN (*n* = 22) and without diabetes (*n* = 22) at a tertiary care center. Patients received an ultrasound-guided axillary block with 30 mL of mepivacaine for anesthesia. The mepivacaine concentration used in each patient was calculated using Dixon’s up-and-down method. A block was considered successful if all four sensory nerves had a score of 1 or 2 within 30 min with no pain during surgery. The primary outcome was the MEAC of mepivacaine, and the secondary outcomes included the minimal nerve stimulation intensity for the musculocutaneous nerve and the occurrence of adverse events. The MEAC_50_ was 0.55% (95% CI 0.33–0.77%) in patients without diabetes and 0.58% (95% CI 0.39–0.77%) in patients with DN (*p* = 0.837). The MEAC_90_ was 0.98% (95% CI 0.54–1.42%) in patients without diabetes and 0.96% (95% CI 0.57–1.35%) in patients with DN (*p* = 0.949). The stimulation threshold for the musculocutaneous nerve was significantly different between groups (0.49 mA vs. 0.19 mA for patients with vs. without diabetes; *p* = 0.002). In conclusion, the MEAC of mepivacaine for a successful axillary block is not lower in patients with DN.

## 1. Introduction

Diabetes is one of the most common chronic conditions and its incidence continues to rise worldwide [1]. Anesthesiologists face challenges when managing patients with diabetes given their pre-existing comorbidities. Compared with other anesthetic techniques, regional anesthesia (RA) offers several advantages, such as hemodynamic stability, reduced opioid use, improved pain relief, decreased postoperative nausea and vomiting, the reduced need for perioperative blood transfusions, and rapid recovery [2,3]. However, a major concern is the potential risk of subsequent nerve injury following RA.

A previous respective study [4] reported that the risk of nerve injury following neuraxial anesthesia or analgesia is 0.4% (95% confidence interval [CI] 0.1–1.3%) in patients with diabetic neuropathy (DN), which is 10-fold higher than the 0.04% in the general population [5]. Several factors, such as anesthetic, patient, and surgical causes, may contribute to this complication.

Local anesthetics (LAs) are potentially neurotoxic in a dose-dependent manner [6]. The nerves in patients with DN may exhibit heightened sensitivity and vulnerability to LA toxicity due to chronic ischemic hypoxia and decreased nerve blood flow [7]. Notably, Gebhard et al. observed a higher block success rate in patients with diabetes, independent of the body mass index [8]. Some studies have also reported that increased sensitivity to LAs is associated with a prolonged block duration [9,10,11]. Thus, the use of a lower LA concentration may decrease the risk of further neurological injury and be beneficial for reducing the total dose required for successful RA [3,12,13]. Previous studies have reported that the LA requirement for a successful block could be lower in nerves with DN, potentially due to the increased sensitivity of nerves to LAs [14]. However, limited information is available regarding the effects of DN on RA outcomes in the upper extremities [9,10].

Therefore, this study aimed to compare the minimum effective anesthetic concentration (MEAC) of mepivacaine for a successful axillary brachial plexus block during upper-extremity surgery between patients with and without DN. We hypothesized that the MEAC of mepivacaine for a successful axillary brachial plexus block would not decrease in patients with DN compared to healthy patients without DN. The primary outcome was the MEAC of mepivacaine, and the secondary outcomes included the minimal intensity of the nerve stimulation of the musculocutaneous nerve and the occurrence of other adverse events (such as LA toxicity, paresthesia, or neurologic deficits). With this study, our goal was to provide evidence that informs clinical practices, fostering a safer and more tailored approach to anesthesia in patients with diabetes. This study holds promise for advancing our understanding of anesthetic considerations in the intricate landscape of DN, thereby enhancing patient care and perioperative outcomes.

## 2. Materials and Methods

### 2.1. Ethics

This study was conducted in accordance with the Declaration of Helsinki and approved by the Institutional Review Board of Inha University Hospital, Republic of Korea, on 13 September 2020 (#2020-09-013-005). This prospective observational study was registered online (https://cris.nih.go.kr/cris/index.jsp, Identifier: KCT0005715, principal investigator: C. Yang, first posted date: 21 December 2020, accesseed date: 1 January 2021), after which patients were recruited from December 2020 to October 2021. All participants received written information about the protocol, had ample time to agree to their participation, and provided written informed consent before enrollment in the study. This study was conducted in accordance with the Strengthening the Reporting of Observational Studies in Epidemiology (STROBE) guidelines [15].

### 2.2. Participants

In this prospective observational study, we recruited consecutive patients with type 2 diabetes scheduled for elective arteriovenous fistula formation surgery and consecutive healthy patients without diabetes undergoing elective orthopedic (involving soft tissue) surgery of the forearm and/or hand. We included patients aged 19–80 years with American Society of Anesthesiologists (ASA) physical status classes I–III. The exclusion criteria were age <19 or >80 years, patient refusal, contraindications to RA (infection and coagulopathy), preexisting neuropathy not attributable to diabetes mellitus, psychiatric history, pregnancy, emergency surgery, alcohol or drug abuse, cardiac or pulmonary decompensation, and allergies to LAs.

The patients were allocated to one of the following two groups: healthy patients without diabetes and without any neuropathy (NDN group), and patients with type 2 diabetes and DN (DN group). A preoperative nerve conduction test was performed in all screened patients in the DN group. Only patients diagnosed with DN by nerve conduction velocity tests were included.

Nerve conduction studies were conducted using Medtronic Keypoint electromyography equipment (Skovlunde, Denmark) in all patients in the DN group. The sensory and motor nerve action potentials were measured using a surface bar electrode (Medtronic 9013L0221, 23 mm). The onset latency, amplitude, velocity, and minimal F-M latency were measured in the ulnar, median (motor and sensory), tibial, superficial, deep peroneal, and sural nerves. The peak latency and peak-to-peak amplitude were determined via a sensory examination of the median, ulnar, superficial peroneal, and sural nerves. DN was diagnosed based on abnormal findings in which a difference of more than two standard deviations (SDs) above the normal value (Table 1) was observed using the method reported by Dyck et al. [16]. DN has been reported to occur after the identification of three or more abnormal findings among onset latency, amplitude, conduction velocity, and F-latency in more than two of the median, ulnar, peroneal, sural, and tibial nerves.

### 2.3. Block Technique

On arrival in the block room, standard monitoring (SpO_2_ measurement, electrocardiography, and non-invasive arterial blood pressure measurement) was performed, and supplemental oxygen was administered throughout the procedure. Midazolam (1–2 mg) was administered intravenously for anxiolysis. An axillary brachial plexus block was performed by an anesthesiologist (C.Y.) using ultrasonography (Viamo c100, Canon Medical Systems, Otawara, Japan) with a 50–13-MHz linear probe. An 80 mm, 22 G insulated needle (UniPlex NanoLine, Pajunk, Geisingen, Germany) and a nerve stimulator (Stimuplex, HNS 12, B. Braun, Melsungen, Germany) were used for all blocks.

The patients were placed in supine position with the shoulder abducted and the elbow flexed. The transducer was placed on the axilla to obtain a short-axis view of the arteries. The needle was initially inserted in-plane toward the axillary artery at the 6 o’clock position and 13 mL of LA was deposited. Subsequently, the needle was withdrawn and advanced to the 12 o’clock position with respect to the axillary artery and 13 mL of LA was deposited. We did not systematically identify the median, radial, or ulnar nerves. Finally, the needle was advanced toward the musculocutaneous nerve and positioned in close contact with the nerve without penetration. With an initial stimulation of 1.0 mA, a stimulation duration of 0.1 ms, and a stimulus frequency of 1 Hz, after the appropriate motor response (elbow flexion), the current was decreased until the motor response vanished and was recorded. At this point, 4 mL of LA was slowly injected. If the musculocutaneous nerve could not be identified, 15 mL of LA was injected at the 6 and 12 o’clock positions. Any adverse events (vessel puncture, LA toxicity, and/or unintentional paresthesia during the block) were recorded.

Mepivacaine has a relatively fast onset and an intermediate block duration. In addition, the surgeries performed in this study mainly involved the soft tissue, resulting in mild pain. Therefore, mepivacaine was chosen as the LA in this study for the axillary brachial plexus block. The study solutions were prepared by an author who was not involved in patient management (N.K.). The patients, anesthesiologists performing the blocks, surgeons, and outcome assessors were blinded to the concentration of mepivacaine used. Based on our clinical experience, the initial mepivacaine concentration in both groups was 1.0%. The mepivacaine concentration used for each patient was determined based on the outcome of the preceding block, using the up-and-down method. When block success was achieved, the concentration used for the following patient was decreased by 0.1%; conversely, for block failure, the concentration in the subsequent patient was increased by 0.1%.

### 2.4. Block Assessment

Block assessment was performed by a blinded investigator (E.J., S.J., or Y.K.) every 5 min for 30 min after LA injection. Sensory block was assessed in the distribution of the radial, median, ulnar, and musculocutaneous nerves using a pinprick test with a 25 G needle on the following scale: 0 = normal sensation, 1 = loss of sensation to pinprick, and 2 = loss of sensation to light touch, compared to the contralateral arm. Block assessment was halted when either block success was achieved or 30 min had elapsed.

Block success was defined as a sensory score of ≥1 in all four target nerves within 30 min regardless of the extent of motor block. Block failure was defined as a sensory score of <1 in any four target nerves at 30 min. In case of block failure, LA was injected at the surgical site and/or opioids were administered, or a switch to general anesthesia was performed, if necessary. Intraoperative sedation was achieved with the continuous infusion of propofol (30–50 μg kg^−1^ min^−2^), if requested by the patient. All patients received intravenous acetaminophen (1000 mg).

Following the operation, the patients were transferred to the post-anesthesia care unit, where their postoperative pain was assessed using a numeric rating scale by post-anesthesia care unit nurses. Patients were evaluated 24 h after surgery for any neurological complications, including paresthesia or motor deficits. The attending surgeon evaluated the patients for neurological deficits in the operated limbs.

### 2.5. Statistical Analysis

To determine the sample size, we assumed that DN would decrease the MEAC of mepivacaine required for a successful axillary block. We considered a 0.4% difference in the LA concentration clinically relevant [17]. Assuming a standard deviation (SD) of 0.45%, a calculated sample size of 16 patients was required for each group, with α = 0.05 and β = 0.2. For possible dropouts, 22 patients were included in each group to avoid dropouts. To calculate the MEAC_50_ using the Dixon and Massey method, a minimum a priori number of five independent negative–positive up-and-down defects is required [18]. We did not use stopping rules to reliably estimate the MEAC_50_. Data are presented as mean ± SD, estimate/standard error (SE), median (range), number (proportion), or 95% CI, as appropriate. The data distribution was evaluated using the Kolmogorov–Smirnov test. The binary data of the block quality (success or failure) were analyzed using logistic regression, with the mepivacaine concentration as the predictor variable, to calculate the MEAC of the LA required to produce a successful block in 50% and 90% of patients (MEAC_50_ and MEAC_90_, respectively). Continuous variables were assessed using Student’s *t*-test or the Mann–Whitney *U* test according to normality. Categorical variables were analyzed using Pearson’s chi-squared or Fisher’s exact test, as appropriate. *p* < 0.05 was considered statistically significant.

## 3. Results

Thirty patients in the NDN group were assessed for eligibility. Of these, eight patients were excluded or refused to participate, and 22 consented to enrollment. A total of 25 patients were assessed for eligibility in the DN group. Of these, three patients were excluded or refused to participate, and 22 consented to enrollment. All patients eligible for inclusion in the DN group were diagnosed with DN after a preoperative nerve conduction test.

**Figure 1:**
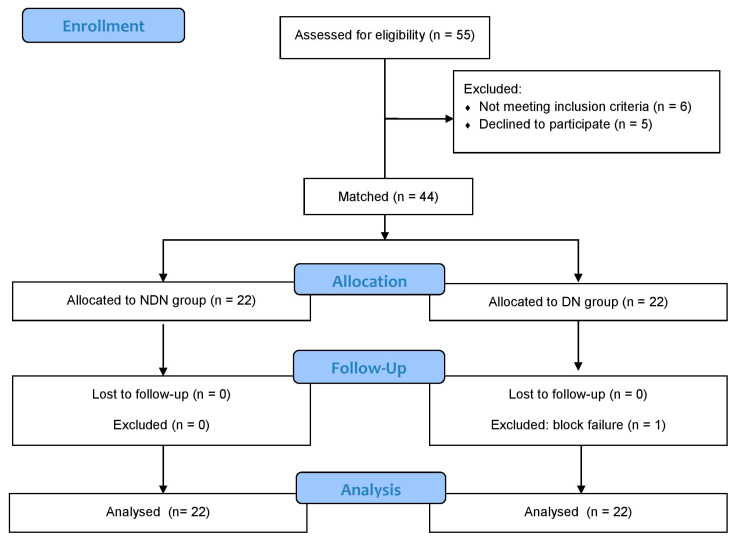
Flowchart of patient selection in this study. NDN, without diabetes and neuropathy; DN, diabetic neuropathy.

Therefore, 44 patients were enrolled in this study (Figure 1). The patient demographics are shown in Table 2. Despite significant differences in age, the ASA physical status class, and serum creatinine levels, the other demographic data were similar between the groups. The surgical duration was shorter in the NDN group than in the DN group. Table 2 presents the characteristics of the nerve conduction tests performed in the DN group.

**Figure 2:**
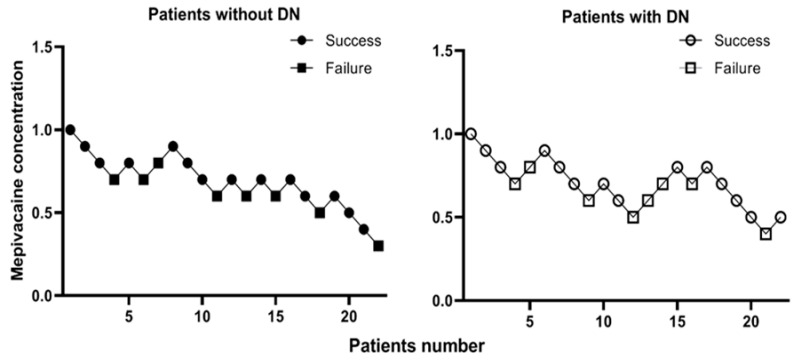
Sequences of successful and unsuccessful blocks in each group. DN, diabetic neuropathy.

The sequences of successful and unsuccessful blocks are shown in Figure 2. The MEAC_50_ of mepivacaine for axillary block was 0.55% (SE 0.11, 95% CI 0.33–0.77%) in the NDN group and 0.58% (SE 0.10, 95% CI 0.39–0.77%) in the DN group (*p* = 0.837). The logistic regression analysis for serum creatinine was 0.86% (SE 0.48, 95% CI, 0–1.8%) in the NDN group and 0.42% (SE 0.25, 95% CI, 0–0.92%) in the DN group (*p* = 0.423). The MEAC_90_ calculated using the logistic regression model was 0.98% (SE 0.23, 95% CI 0.54–1.42%) in the NDN group and 0.96% (SE 0.20, 95% CI 0.57–1.35%) in the DN group (*p* = 0.949). The logistic regression analysis for serum creatinine was 1.32% (SE 0.65, 95% CI 0.04–2.60%) in the NDN group and 0.77% (SE 0.25, 95% CI 0.29–1.26%) in the DN group (*p* = 0.438).

Eight patients in each group had an unsuccessful block. Four and six patients with unsuccessful blocks in the NDN and DN groups required intravenous fentanyl intraoperatively, respectively. Among the patients with block failure, one patient in both groups required intravenous fentanyl because of postoperative pain in the post-anesthesia care unit.

The minimal intensity of nerve stimulation for the musculocutaneous nerve was significantly lower in the NDN group (0.19 [0.08] mA) than in the DN group (0.49 [0.15] mA, *p* = 0.002). No adverse events associated with the blocking procedure were observed. No neurological deficits were observed in either group 24 h after surgery.

## 4. Discussion

In this study, we compared the MEAC of mepivacaine for successful axillary brachial plexus block during upper-extremity surgery between patients with and without DN. We found that patients with DN did not show a significant decrease in the MEAC of mepivacaine for successful axillary brachial plexus block compared to patients without neuropathy. However, a significant difference was found in the minimal intensity of the nerve stimulation of the musculocutaneous nerve between the two groups.

Whether DN increases the risk of nerve injury following RA remains controversial. In animal studies, LA-induced nerve injury was observed in rats with DN [19]. With the potential risk of secondary neurological injury in patients with DN due to the double-crush phenomenon, the use of a lower concentration (reduced dose) of LA is recommended [3,12,13]. A previous animal study showed that the nerve fibers of rats with DN are more susceptible to LA action and that the amount of LA required for a successful sciatic nerve block is lower in rats with DN than in control rats [14]. However, we observed no significant difference in the MEAC of mepivacaine for a successful axillary block between the two groups in this study. This discrepancy may be explained by the difference in the severity of DN between the upper and lower extremities. DN is more severe in the lower extremities than in the upper extremities, and the distal nerves are involved before the proximal parts [20]. In this study, the DN group showed nearly normal findings in the nerve conduction studies, as shown in Table 1 and Table 3. Thus, the criteria by Dyck et al. for DN may not exclude the relatively well-preserved function of the median/ulnar nerve of the upper extremity in patients with DN.

Another factor affecting our findings may be the LA volume in this study. A relatively large volume (30 mL) of mepivacaine was used for axillary brachial plexus block. This may have obscured the differences in concentrations between groups. A perineural technique in which the four nerves are separately localized and anesthetized can yield different results.

Our study suggests that nerves of the upper extremities in patients with DN might not have increased sensitivity to LA compared to those of the lower extremities. Thus, regional anesthesia for upper-extremity surgery in patients with DN may not require a reduced dose of LA for a successful block. However, previous studies observed an increased block duration with reduced analgesic consumption following upper-extremity peripheral nerve block [9,10], which might be related to an increased sensitivity to LA in patients with DN. Further large-scale confirmatory studies are required.

Consistent with previous studies [21,22], this study showed a significant difference in the minimum intensity of the nerve stimulation of the musculocutaneous nerve between the two groups. This suggests that nerve fibers in the upper extremities of patients with diabetes could be damaged, similar to those in the lower extremities, although to a lesser degree. Thus, only stimulation-guided peripheral nerve block may increase the risk of intraneural injection. Ultrasound guidance may help to avoid this complication. However, other studies have reported no significant differences between patients with and without diabetes [23,24]. This may be explained by the fact that DN, but not diabetes, can lead to altered nerve excitability. Unfortunately, we did not assess the stimulation thresholds of the other three nerves of the upper extremities in this study. Therefore, our findings may not apply to other peripheral nerves in the upper or lower extremities.

This study had some limitations. First, we used a classic up-and-down design for this dose-finding study, which might not appropriately determine the clinically relevant dose for 90% of the participants [25]. Second, due to our study design, we used a relatively small sample. A larger sample size would increase the reliability and generalizability of these findings. Third, the healthy controls did not undergo preoperative nerve conduction tests, indicating that subclinical neuropathy may have affected our results. Fourth, the definition of block success in this study was based only on the sensory blocks. In addition, because of our block techniques, block failure due to technical reasons rather than the LA effect cannot be excluded. Fifth, we did not evaluate postoperative outcomes such as the block duration, pain score, or opioid consumption. Finally, owing to several limitations, our data may not be suitable for generalization to other types of LAs. These limitations warrant caution in interpreting our findings and highlight avenues for future research to refine our understanding of the intricate interplay between DN and RA outcomes.

Nevertheless, this study provides valuable insights into the intersection of the DN and the RA, shedding light on the MEAC of mepivacaine in axillary brachial plexus blocks. The meticulous use of a prospective observational design, the inclusion of both diabetic and nondiabetic patients, and reliance on ultrasonography for guidance enhanced the internal validity of this study. Clinically, these findings have implications for anesthesiologists in navigating the intricate balance of achieving optimal block success while minimizing the risk of nerve injury in patients with diabetes.

## 5. Conclusions

In conclusion, our investigation of the impact of DN on the MEAC of mepivacaine for axillary brachial plexus blocks challenges the conventional assumptions derived from animal studies. DN did not reduce the MEAC of mepivacaine for successful axillary brachial plexus blocks. The observed differences in the nerve stimulation intensity for the musculocutaneous nerve between patients with and without diabetes emphasize the need for tailored approaches to RA. Further exploration of the distinct effects of DN on anesthesia outcomes could contribute to the development of targeted approaches in clinical practice.

## Figures and Tables

**Table 1:** Electrophysiological criteria for abnormal nerve conduction study.

Median Motor Nerve	Median Sensory Nerve	Ulnar Motor Nerve	Ulnar Sensory Nerve
L > 4.0 ms	L > 3.5 ms	L > 3.8 ms	L > 3.4 ms
A < 5.0 ms	A < 1.0 μV	A < 5.0 mV	A < 7.5 μV
CV < 49.0 m/s		CV < 49.0 m/s	
MF > 24.2 ms		MF > 24.8 ms	
Peroneal motor nerve	Superficial peroneal sensory nerve	Tibial motor nerve	Sural sensory nerve
L > 4.5 ms	L > 3.5 ms	L > 5.0 ms	L > 3.5 ms
A < 1.0 mV	A < 3.7 μV	A < 5.0 mV	A < 5.0 μV
CV < 40.0 m/s		CV < 40.0 m/s	
MF > 45.0 ms		MF > 45.3 ms	

A, amplitude; CV, conduction velocity; L, latency; MF, minimal F-M latency.

**Table 2:** Patient demographics and characteristics.

Variable	No Diabetic Neuropathy Group (*n* = 22)	Diabetic Neuropathy Group (*n* = 22)	*p* Value
Age (years)	45 ± 18	60 ± 11	0.003
Sex (male/female)	12/10	12/10	1.000
Height (cm)	167 ± 11	163 ± 10	0.949
Weight (kg)	71 ± 15	66 ± 15	0.743
Body mass index (kg m^−2^)	25 ± 4	25 ± 4	0.889
ASA physical status class (I/II/III)	11/10/1	0/0/22	<0.001
Surgical time (min)	46 ± 29	89 ± 43	<0.001
History of diabetes (years)	NA	16 ± 2	
Insulin use	NA	7	
Oral hypoglycemic agent	NA	15	
Hemoglobin A1c (%)	NA	7.5 ± 1.6	
Serum creatinine (mg/dL)	0.81 ± 0.18	6.60 ± 2.50	<0.001

Data are presented as mean ± SD or *n*. ASA, American Society of Anesthesiologists; SD, standard deviation.

**Table 3:** Electrophysiological characteristics of patients with diabetic neuropathy.

Nerve	Latency (ms)	Amplitude (mV)	Conduction Velocity (m/s)	Minimal F-M Latency (ms)
Median sensory nerve	3.6 ± 0.8	13.8 ± 16.6	NA	NA
Motor nerve	4.8 ± 1.6	5.0 ± 3.6	44.5 ± 6.4	30.4 ± 5.5
Ulnar sensory nerve	4.5 ± 1.2	11.9 ± 18.3	NA	NA
Motor nerve	3.7 ± 1.6	5.1 ± 3.1	45.1 ± 9.7	30.2 ± 5.2

Values are presented as mean ± standard deviation.

## Data Availability

The data presented in this study are available upon request from the corresponding author.
